# Further validation of the Chinese short Warwick Edinburgh mental wellbeing scale in the adult population of Macau: an application of classic test theory and item response theory

**DOI:** 10.3389/fpsyt.2025.1528509

**Published:** 2025-02-26

**Authors:** Lawrence T. Lam, Mary K. Lam

**Affiliations:** ^1^ Faculty of Medicine, Macau University of Science and Technology, Macao, Macao SAR, China; ^2^ Faculty of Medicine and Health, The University of Sydney, Sydney, NSW, Australia; ^3^ Faculty of Health, University of Technology Sydney, Sydney, NSW, Australia; ^4^ Department of Health and Biomedical Sciences, RMIT University, Melbourne, VIC, Australia

**Keywords:** SWEMWBS, mental well-being, classic test theory, IRT, Chinese

## Abstract

**Background:**

This study aims to validate the Chinese version of the Short Warwick-Edinburgh Mental Well-being Scale (SWEMWBS) by employing both Classical Test Theory (CTT) and Item Response Theory (IRT) approaches.

**Methods:**

Data were gathered through a population-based, cross-sectional health survey using an online self-reported questionnaire. The scale underwent Exploratory Factor Analysis (EFA) and Confirmatory Factor Analysis (CFA). Measurement invariance by gender was assessed using standard procedures. The Grade Response Model (GRM) of the IRT analysis was applied to the data, estimating the discrimination and difficulty parameters at different thresholds. The results were analyzed both graphically and through parameter values.

**Results:**

Factor analyses confirmed that a single-factor model of the scale fit the data well, with an overall Eigenvalue of 4.55, explaining 65.0% of the total variance. Model fit statistics were slightly better for males than for females. Measurement invariance examinations also yielded satisfactory Goodness-of-Fit statistics (CFI = 0.940, TFI = 0.910, RMSEA < 0.001) with minimal changes in item loadings and indicator threshold patterns across groups. The IRT results demonstrated high discrimination parameters, ranging from 2.17 to 3.67, and nearly evenly distributed difficulty parameters, ranging from -2.23 to 1.77. Graphical examinations indicated good performance of the scale across the latent trait continuum.

**Conclusions:**

The results indicated that, as a single-factor scale, the instrument exhibits good quality at both the scale and item levels. It has high discriminative power and an adequate response set for assessing a full range of the latent trait, namely mental well-being.

## Introduction

Mental well-being, often synonymous with positive mental health, encompasses a multifaceted understanding of self-acceptance, personal growth, resilience, autonomy, and mastery of one’s environment (1). Rather than solely focusing on mental illness, the World Health Organization (WHO) increasingly emphasizes the significance of positive mental health for overall population well-being (2). Over the past two decades, mental well-being has garnered substantial attention. The WHO defines positive mental health as a “state of well-being in which individuals recognize their own abilities, effectively cope with life’s normal stresses, contribute productively, and engage with their community” (3). This definition underscores that mental health extends beyond the mere absence of illness (4).

Based on the initial concept of mental well-being proposed by scholars such as Jahoda, Kayes et al., Waterman et al., and others (1, 5–8), Tennant and colleagues proposed a two-dimensional model of mental well-being consisting of hedonic and eudaimonic aspects (9, 10). The hedonic aspect refers to the individual’s subjective feelings of happiness and life satisfaction, whereas the eudaimonic aspect relates to psychological functioning and the actualization of an individual’s potential, capacity, and positive relationship with self and others. These early models significantly influenced the development and validation of the Warwick-Edinburgh Mental Well-being Scale (WEMWBS) (9, 10).

Since the scale’s publication in the UK, the WEMWBS has been translated into many languages and used in various countries and regions. Interested readers can visit the official website for a list of translations (https://warwick.ac.uk/fac/sci/med/research/platform/wemwbs/using/translations/). Numerous validation studies have been conducted on both the original 14-item scale (11–16) and the 7-item Short Warwick-Edinburgh Mental Well-being Scale (SWEMWBS) (17–28). The Chinese version of the SWEMWBS was first translated by Ng et al. in 2014 (17) and has been used in several studies since then (21–23, 29–33).

In terms of validating the SWEMWBS, many studies have utilized classical psychometric evaluations to examine reliability and validity, including factor analyses for structural validity and conventional techniques for convergent and divergent validity (20, 23, 25, 26, 34–36). Some validation studies also included measurement invariance assessments to provide further evidence for the scale’s validity (20, 34, 35). The exploratory and confirmatory factor analysis results of most studies affirmed that a single-factor model with global goodness-of-fit indices fit best to the data (20, 23, 25, 26, 34). Reliability assessments indicated reasonable reliability for the single-factor scale, with Cronbach’s alpha values ranging from 0.77 to 0.88 (20, 26). Sarasjärvi et al. reported a reliability measure of McDonald’s omega at 0.92 (25). For convergent validity, Ng et al. found moderate correlations between the scale and the General Health Questionnaire (GHQ-12) (r = -0.52, p < 0.001), Happiness Index (r = 0.53, p < 0.001), and EQ-VAS (r = 0.40, p < 0.001) (23). Koushede et al. reported moderate to high correlations with WHO-5 (r = 0.75, p < 0.05), Self-rated Health (r = 0.48, p < 0.05), PHQ-4 (r = -0.58, p < 0.05), and PSS (r = 0.65, p < 0.05) (20). Melendez-Torres et al. found correlations with Life Satisfaction (r = 0.50, p < 0.001), Somatization (r = -0.53, p < 0.001), School Pressure (r = -0.30, p < 0.001), and Bullying Victimization (r = -0.30, p < 0.001) (35). More recently, Sarasjärvi et al. found high correlations with GHQ-12 (r = -0.758, p < 0.001), MHI-5 (r = 0.731, p < 0.001), BDI-6 (r = -0.866, p < 0.001), and EUROHIS-QOL8 (r = 0.718, p < 0.001) (25). However, low to moderate correlations were found between SWEMWBS items and Need Satisfaction (r = 0.544, p < 0.001), Need Frustration (r = -0.359, p < 0.001), and Depression (r = -0.33, p < 0.001) in Zayed et al.’s study (26). Criterion validity was assessed in some studies (20, 33). For example, Ng et al. found that participants with lower self-rated health were more likely to have lower than medium well-being (OR = 9.1, 95% CI = 8.05-11.23) (20).

The Rasch Model and Item Response Theory (IRT) have been used as alternative approaches for validating WEMWBS for a long time (37, 38). These methods have become more frequent in recent validation studies on SWEMWBS (14, 28, 39). Results from these studies suggest that all items have high discrimination ability with relatively large discrimination parameters. For difficulty parameters, the scale appears to measure and discriminate respondents better with a negative latent trait, indicating a lower level of mental well-being (28, 39). Overall, the scale showed good functioning along the latent trait continuum, particularly within the range of about -2.0 to 2.0 (28, 39).

Few studies have employed both Classical Test Theory (CTT) and IRT to examine the structural validity of the SWEMWBS using factor analyses. Smith et al. evaluated the scale with factorial analyses and also utilized the Graded Response Model (GRM) of IRT to examine age differences in the latent factor (Theta). No statistically significant differences were found between middle-aged and young adults (14).

For the Chinese SWEMWBS, of the eight studies identified in the literature (17, 21–23, 29–32), four validation studies employed the classical approach (17, 21–23). These studies utilized samples based in a single location, namely Hong Kong, except for one conducted in China. One study involved patients with mental illness (17), another university students (21), and the China study utilized small samples of healthy participants and patients with chronic diseases (33). Sun et al. conducted the only study on the general population (22). To the author’s knowledge, no study has been conducted on the Chinese SWEMWBS in the community employing both classical psychometric evaluation and IRT. Hence, this study aims to further validate the Chinese SWEMWBS using a community sample of Macau residents and adopting both Classical Test Theory, particularly factor analysis and measurement invariance, and IRT approaches.

## Methods

### Sample and data collection

Data utilized in this study was collected from a population-based cross-sectional health survey using a self-reported online questionnaire. The survey was conducted between April and July 2024 among adult residents in Macau. The online questionnaire was distributed through 23 associations and societies involved in the city-wide survey as collaborating partners. These collaborating partners represented many different natures of groupings, with some being professional bodies, others being community associations, and some non-government organizations. With the support of the management of these organizations, members were encouraged to participate in the survey through public appeals and personal invitations. The total number of potential participants was more than 50,000, accounting for nearly 9% of the adult population in Macau. The sample consisted of 1460 respondents, with 1001 females (68.6%) and nearly 45% in the age group of 18-34 years (n=655, 44.9%). Ethics approval was obtained from the Faculty Ethics Research Committee of the Faculty of Medicine, Macau University of Science and Technology (MUST-FMD-200402025001).

### Measure

#### The short form of the Warwick-Edinburgh mental well-being scale

The 14-item WEMWBS was validated and widely used in many mental well-being studies. It had good content and structural validity with a single factor demonstrated by Confirmatory factor analysis (9). It also showed high reliability, with Cronbach’s alpha scores ranging from 0.89 to 0.91. WEMWBS correlated strongly with other mental health and well-being scales and lower correlations with scales measuring overall health with a test-retest reliability of 0.83 at one week (9). The Short Warwick Edinburgh Mental Wellbeing Scale (SWEMWBS). (SWEMWBS © NHS Health Scotland, University of Warwick and University of Edinburgh, 2008, all rights reserved) was derived from the original scale with 7 items by Stewart-Brown et al. in 2009 (37). As with the full scale, the short form was also designed as a scale with a 5-point frequency Likert response set ranging from 1=rarely to 5=all the time, resulting in a minimum total score of 7 and a maximum of 35. More information on the SWEMWBS can be found on the official website. (https://www.corc.uk.net/outcome-experience-measures/short-warwick-edinburgh-mental-wellbeing-scale-swemwbs/) The authors recommended that the raw scores of the SWEMWBS should be converted to the provided corresponding matric scores before data analysis and interpretation.

### Data analysis

Data were analyzed using the STATA statistical software (StataNow 18.5). For structural validity, the scale was subjected to Exploratory Factor Analysis (EFA) and Confirmatory Factor Analyses (CFA) with two separate subsets of data, the test and the validation datasets, generated randomly and evenly from the total dataset. To determine whether the 7-item scale was suitable for factor analysis, Barlette’s test for sphericity and the Kaiser-Meyer-Olkin (KMO) measure of sample adequacy were used. Upon the satisfaction of these examinations, EFA was conducted using principal component factors with orthogonal varimax rotation as the extraction method. The factor model was examined using the Eigenvalue (>1.0) and the overall model statistics with a Likelihood Ratio test. CFA was carried out on the validation dataset to further examine the factor structure of the scale. Structural Equation Modelling (SEM) was used as the analytical approach. The maximum likelihood estimation for covariance structure analysis was used to examine the factorial structure after testing for multivariate normality. Results indicated that the assumption of multivariate normality was not violated (Mardia mSkewness, Mardia mKurtosis, and Henze-Zirkler tests p>0.05). The goodness of fit of the model to the data was determined using multiple criteria, including the Chi-square value (χ^2^, p>0.05), the Comparative Fit Index (CFI, >0.90), the Root Mean-Square Error of Approximation (RMSEA, app. = 0.06), the Tucker–Lewis index (TLI, >0.90), the Standardized root mean squared residual (SRMR, <0.08), and the Akaike’s information criterion (AIC) and Bayesian information criterion (BIC), as suggested by Byrne (36). The factor loading of each item was also calculated and reported using the path diagram. Upon confirming the unidimensional structure of the scale, the scale was subjected to further examinations of the measurement invariance properties. The standard procedures of the measurement invariance were applied to ascertain whether there were any differences in the factor structure and the measurement between gender groups. Four different models were fitted to the data to examine the hierarchy of factors invariance, with model 1 being the configurational model, which is the less restrictive model. Model 2, the loadings model, and Model 3, the loadings and thresholds, were then fitted and followed by the additive model, including the loading, threshold, and residual as Model 4. At each step, the invariance test was conducted to compare between models to determine whether the fitted model was worse off than the previous model. As noted by Anthony et al., there was a lack of agreement in the indices of the invariance test for categorical and ordinal data; the decision to compare the model could only be based on the current recommendation (34). Adopting a similar approach, Cheung et al. recommended that a decrement in the CFI or RMSEA by a magnitude of -0.01 in the model in comparison to the previous model suggested minimal measurement variance (40).

In terms of the IRT approach, while agreeing with the view of Hanzlová et al. that the response set of the items is polytomous and ranked, thus the Grade Response Model (GRM) of the IRT analysis is an appropriate approach (28). As defined by the GRM, two main parameters were estimated, namely the discriminant and the difficulty parameters at different thresholds (refers to the latent trait and symbolized by theta θ). The discriminant parameter refers to how well the item could be used to distinguish respondents with different levels of the latent trait. In the current analysis, four difficulty parameters were estimated for each item since there were 5 responses in the response set (4 ranks). In the GRM, the difficulty parameters of different thresholds could be understood as a 50% chance that the respondent would choose a specific response or higher on the scale (e.g. choosing sometimes, often, or all the time). For determining the acceptable values of these parameters, it was suggested that a value within the range between 0 and ≥ 2 and -2 to 2 for discriminant and difficulty, respectively (41). The range of discrimination parameter values and the interpretation were listed in the Supplementary Information. For assessing whether the GRM fitted well with the data, the results were examined by the values of the parameters and graphically including the Item Characteristics Curves (ICC), Category Characteristics Curves (CCC), Item Information Functions (IIF), and the Test Information Function (TIF). Similar to the measurement invariance examination in the factor analysis, the GRM was also fitted by gender groups to investigate for any difference in the item responses between males and females.

## Results

### Factor analysis

**Figure 1:**
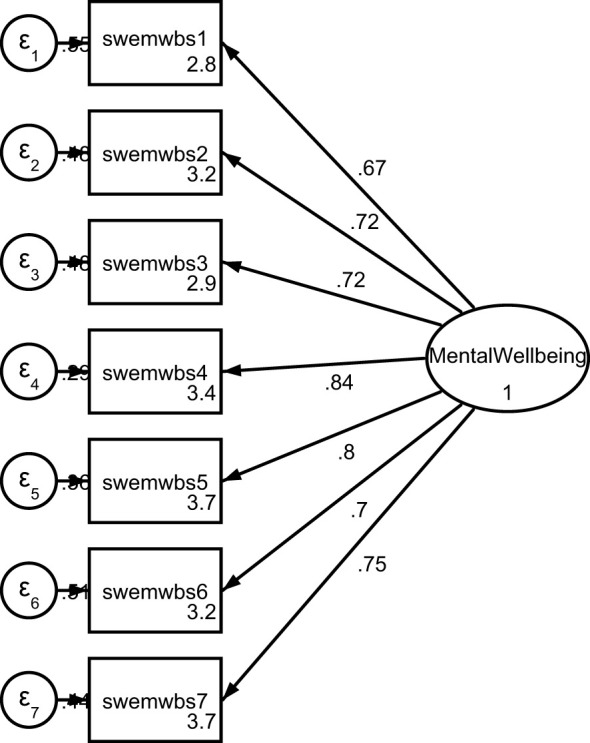
Path diagram of the confirmatory factor analysis.

Before conducting the Exploratory Factor Analysis (EFA), the sample was randomly split into two equal datasets, resulting in 730 data points for both the testing and confirmatory datasets. To assess the suitability of the testing data for factor analysis, the Bartlett test of sphericity and the Kaiser-Meyer-Olkin (KMO) Measure of Sampling Adequacy were applied, yielding results of χ² (21) = 3016.271, p < 0.0001 and KMO = 0.908, respectively. These results suggested that factor analyses were appropriate for analyzing these data. The EFA results indicated that a single-factor model fit the data well, with an Eigenvalue of 4.55, explaining 65.0% of the total variance. Separate analyses by sex resulted in slightly better model fits for males than females, with Eigenvalues of 4.74 and 4.44, and 67.7% and 63.4% of the total variance explained, respectively (**Table 1**). The factor structure was further examined using Confirmatory Factor Analysis (CFA) with the confirmatory dataset. As summarized in **Table 1**, the Goodness of Fit statistics suggested a good fit of the single-factor model to the data, with all statistics meeting the criteria except the RMSEA, which was slightly larger than the acceptable value for a good fit. The factor loadings of the items ranged from moderately high to high (0.673 to 0.843). The loadings for females were generally lower than those for males, with slight deviations in model fit statistics. The path diagram of the CFA is depicted in **Figure 1**.

**Table 1:** Results of the exploratory and confirmatory factor analyses.

Item	Description			Factor loading		
				Full	Male	Female
1	I’ve been feeling optimistic about the future			0.673	0.647	0.686
2	I’ve been feeling useful			0.722	0.719	0.725
3	I’ve been feeling relaxed			0.721	0.727	0.723
4	I’ve been dealing with problems well			0.843	0.895	0.809
5	I’ve been thinking clearly			0.802	0.841	0.774
6	I’ve been feeling close to other people			0.701	0.750	0.668
7	I’ve been able to make up my own mind about things			0.746	0.761	0.735
Eigenvalue				4.55	4.74	4.44
Total Variance explained				65.0%	67.7%	63.4%
Goodness of Fit statistics	CFI	RMSEA (95%C.I.)	TLI	SRMR	AIC	BIC
Full scale	0.930	0.137 (0.121-0.154) P<0.001	0.894	0.047	12091.005	12187.459
Male	0.935	0.140 (0.111-0.171) p<0.001	0.903	0.050	4048.581	4120.963
Female	0.926	0.135 (0.115-0.156) P<0.001	0.889	0.047	8035.68	8124.10

Measurement invariance of the scale was examined by fitting four different models with increasing constraints by sex. The results are presented in **Table 2**. The configurational (baseline) model demonstrated an acceptable fit to the data with reasonably satisfactory Goodness-of-Fit statistics (CFI = 0.940, TFI = 0.910, RMSEA < 0.001), although the RMSEA was slightly inflated. This suggested that the constructs were similar between the male and female groups regarding free and fixed loadings. When more restrictive models were fitted, the Goodness-of-Fit statistics revealed minimal changes with increasing model restrictions. As shown in **Table 2**, the changes in CFI were less than -0.01 across models except for the additive model. Similar patterns of changes were observed for the RMSEA values. These results suggested that the item loadings and indicator thresholds exhibited similar patterns across groups.

**Table 2:** Measurement invariance tests of the SWEMWBS by sex.

Model constraints	CFI	RMSEA (95%C.I.)	Change in CFI	Change in RMSEA
Configurational	0.940	0.130 (0.118-0.143)	–	–
Loadings	0.939	0.119 (0.108-0.130)	-0.001	-0.011
Loadings, thresholds	0.936	0.111 (0.102-0.131)	-0.003	-0.008
Additive	0.924	0.096 (0.087-0.105)	-0.012	-0.015

### IRT analysis

**Figure 2:**
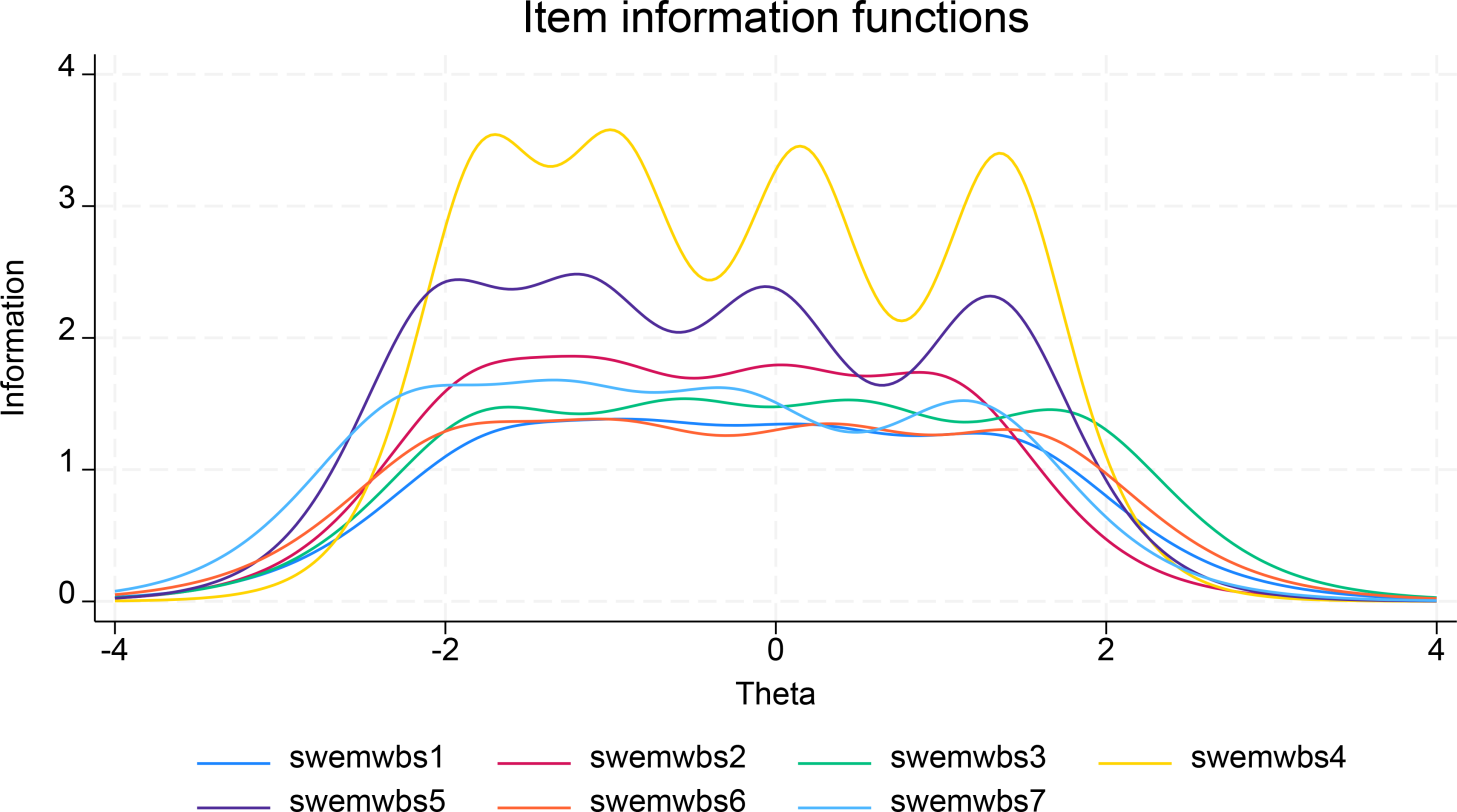
Item Information Functions (IFF) of the scale.

The discrimination and difficulty parameters at different thresholds were estimated using the Graded Response Model (GRM) of Item Response Theory (IRT) analysis. The results for these parameters across the full dataset are summarized in **Table 3**. The discrimination parameters for all items were high, with values greater than 2, ranging from 2.17 for item 1 to 3.67 for item 4 (**Table 3**). This indicated that the items had good discriminating ability to distinguish respondents with different latent trait levels. These results were further verified by the Item Information Functions (IIFs) (**Figure 2**), showing item 1 at the lowest position with the lowest discrimination parameter value, containing less information as exhibited by a flat curve. In contrast, item 4 had the highest discriminating value, occupying the highest position in the graph and containing more information as reflected in the multiple wave shapes, indicating variability in the curve (**Figure 2**).

**Table 3:** Discriminant and difficulty parameters obtained from the GRM for the full sample and by sex.

	Discriminant parameters		Difficulty parameters at each threshold							
			≥ 2		≥ 3		≥ 4		= 5	
Item 1	2.17		-1.71		-0.81		0.22		1.40	
Item 2	2.54		-1.83		-1.03		0.02		1.04	
Item 3	2.35		-1.76		-0.59		0.51		1.77	
Item 4	3.67		-1.77		-0.96		0.15		1.37	
Item 5	3.02		-2.04		-1.14		-0.03		1.32	
Item 6	2.21		-1.95		-0.93		0.32		1.55	
Item 7	2.43		-2.23		-1.28		-0.23		1.19	
	Discriminant parameters		Difficulty parameters at each threshold							
			≥ 2		≥ 3		≥ 4		= 5	
	M	F	M	F	M	F	M	F	M	F
Item 1	2.19	1.63	-1.41	-2.37	-0.62	-1.09	0.32	0.34	1.34	2.02
Item 2	2.57	1.92	-1.53	-2.52	-0.77	-1.44	0.19	0.02	1.10	1.44
Item 3	2.68	1.66	-1.53	-2.38	-0.43	-0.78	0.50	0.79	1.52	2.63
Item 4	4.23	2.60	-1.51	-2.41	-0.77	-1.29	0.18	0.29	1.20	2.05
Item 5	3.35	2.19	-1.68	-2.86	-0.87	-1.60	-0.07	0.08	1.08	2.03
Item 6	2.55	1.53	-1.50	-2.87	-0.68	-1.32	0.32	0.53	1.35	2.33
Item 7	2.59	1.79	-1.81	-3.19	-0.97	-1.82	-0.06	-0.32	0.99	1.84

**Figure 3:**
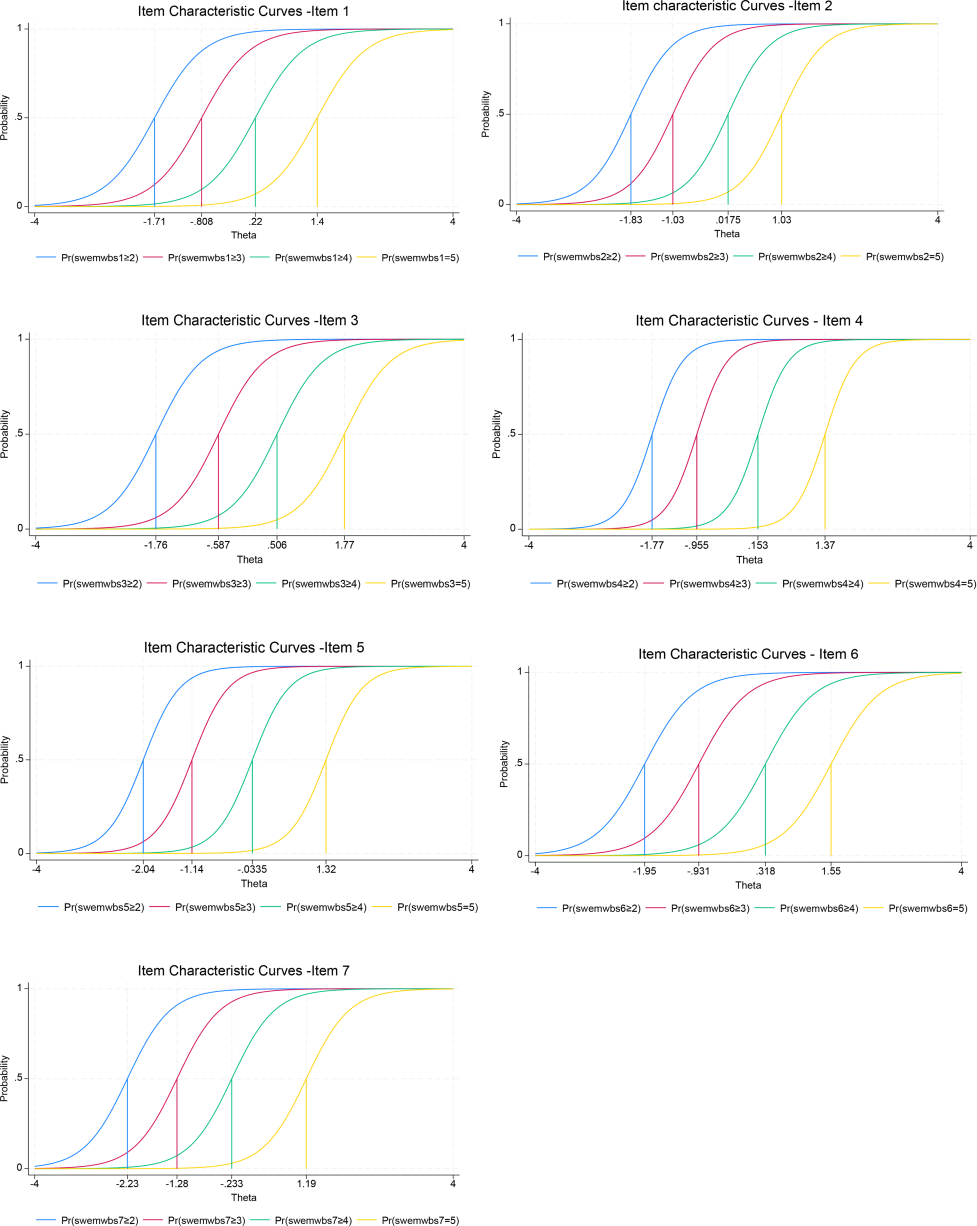
Item Characteristics Curves (ICC) of each item.

Regarding the difficulty parameters for different thresholds, of the 28 parameter estimates, ranging from -2.23 for item 7 to 1.77 for item 3 (**Table 3**), slightly more than half (16, 57%) were negative, and 12 were positive. This suggested that these items could measure and distinguish respondents with negative latent traits (mental well-being) slightly better than those with positive traits. However, the differences were not significant. Some differences in the difficulty parameters at various thresholds were observed between and within items. The difference between items reflected that individuals endorsing the same category threshold for different items would have different degrees of the latent trait. The differences in the difficulty parameters within each item also varied across all 7 items. For instance, the differences in item 1 were 0.90, 1.03, and 1.18, whereas the differences in item 7 were 0.95, 1.05, and 1.42. This suggested that item 1 provided slightly better functioning than item 7. The differences in difficulty parameters were lowest in item 2, with values of 0.80, 1.05, and 1.02. These results were supported by the Item Characteristic Curves (ICCs), which show various item characteristics in **Figure 3**.

**Figure 4:**
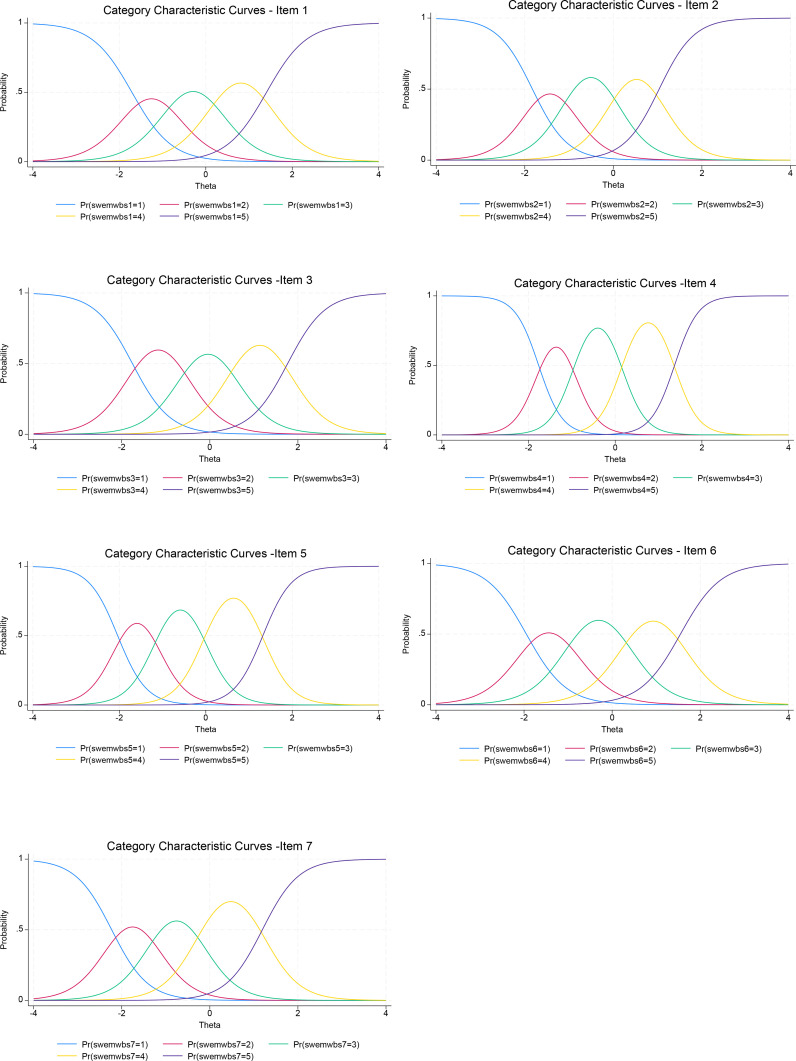
Category Characteristics Curves (CCC) of each item.

**Figure 5:**
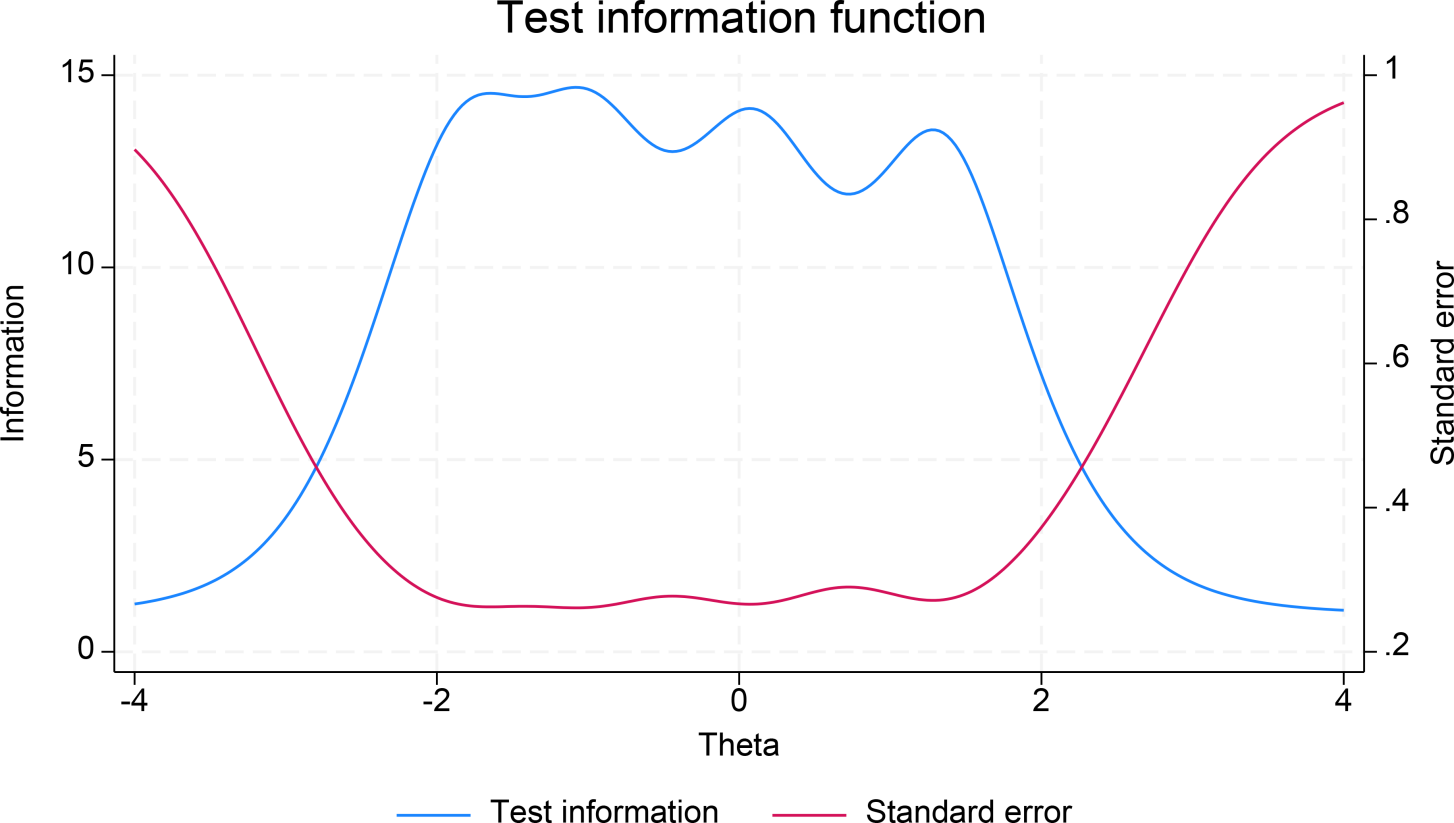
Test Information Function (TIF) of the scale.

The results depicted in the Category Characteristics Curves (CCCs) provided additional information on the scale’s functioning in terms of item responses (**Figure 4**). Each item’s plot exhibited distinct peaks along the latent trait spectrum without closely overlapping or superimposing on each other. This suggested that each response category had its probability distribution at all levels of the latent trait. **Figure 4** displayed a satisfactory set of CCCs for these items, with items 4 and 5 having sharper peaks than the other items. For the full scale, the Test Information Function (TIF) graph indicated good performance across the entire latent trait range, particularly within the range of -2.0 to about 1.8 of the theta value, with minimal standard errors (**Figure 5**). The Test Characteristics Curve (TCC) also provided the predicted scores of 10.8, 23.5, and 33.5 corresponding to -1.96, 0, and 1.96 of the theta values (**Supplementary Figure S1** in supplementary materials).

Similar to the factor analysis, the IRT analysis by gender was also conducted, with results reported in **Table 3** and **Supplementary Figures S2**, **S3**, **S4** in the supplementary materials. As shown, there were differences in the discrimination and difficulty parameters at different thresholds between male and female respondents (**Table 3**). Overall, the discrimination parameter estimates for males were larger than those for females, particularly for item 4, with values of 4.23 for males and 2.60 for females. Differences in the difficulty parameters at different thresholds were also observed, with larger values for males on the negative latent trait than for females, but larger values for females on the positive latent trait than for males. This suggested differences in responses to the scale items between sexes along the latent trait continuum. These differences were also reflected in the results obtained from the TIF and TCC (**Supplementary Figures S3**, **S4**), although the CCC did not show significant differences between groups (**Supplementary Figure S2**).

## Discussions

The growing importance of mental well-being as a global indication of mental healthiness addresses the balance between the negative focuses on mental health problems and illnesses and the positive development of mental wellness. The availability of a well-constructed and validated instrument for the measurement and assessment of the well-defined construct of mental well-being is of paramount importance for the ongoing development of research and clinical practice in the discipline of mental public health.

The SWEMWBS, as one of the few instruments designed specifically for the assessment of mental well-being, has been properly translated into different languages. With the growing development in the field of mental well-being research, there is also an increasing demand for the scale to be used as the outcome measure of research and practice globally. Hence, more substantiating evidence on the reliability and validity of different language versions of the scale will certainly enhance the confidence of researchers and practitioners in the adaptation and usage of the scale. In recent years, an emerging trend in the interest of mental well-being, particularly intervention programs in improving the mental well-being of young people, in the East Asia region has been observed (42) The availability of a theoretically-based and well-designed scale with strong substantiating evidence in the psychometric properties for assessing mental well-being in the Chinese and other East Asian languages will further motivate research in this field.

This study is the first to employ both CTT and IRT approaches for validating the Chinese SWEMWBS. The factor loadings and the measurement invariance results of the factor analyses in this study were comparable to those obtained from the studies by Anthony et al. and Melendez-Torres et al. (34, 35). In terms of the model fit statistics, particularly the greater than the expected values of the RMSEA (0.06) in the full model and the models for males and females, a possible explanation is that there would be a few larger residuals involved that might have skewed the calculation resulting in a larger value. This phenomenon is worthy of more investigation, given that all other goodness-of-fit statistics indicated a reasonably good fit of the model to the data. Another possible explanation is that some other models, such as a two-factor model, may provide a better fit to the data. Considering the overall evidence provided by the model statistics, in conjunction with the volume of evidence from the literature, the single model is still the best option. For the measurement invariance, the results of the current study echoed those obtained by the two reports. In this study, the changes in CFI and RMSEA were less than or close to -0.01 across models. These results were similar to that obtained by Anthony et al. (34). These suggested that the model fits did not change significantly from the most unrestricted configural model to more restricted invariance Metric and Scalar models demonstrating the measure invariance between groups.

The IRT GRM provides further evidence for the scale’s validity with large values of discrimination parameters demonstrating the high discriminative ability of all items, particularly item 4. The difficulty parameters at each threshold also showed good performance of all items along the latent trait continuum. The five-category response set was also shown to be appropriate and adequate, as well as performed well based on the results of the CCC. Different peaks were shown clearly in the curves for all items. For the whole test, the functioning of the scale was demonstrated to provide good information in the TIF graph. In comparison to the study by Hanzlová among adolescents in the UK, these results were comparable and consistent (39). In contrast to the few validation studies of SWEMWBS identified in the literature using the IRT approach, further examinations of the scale by GRM with different gender groups were performed. The results indicated slight differences in the discriminative power of the scale between males and females in favor of the male counterpart. Similarly, differences were also found in the difficulty parameters at each threshold with larger values for males on the negative latent trait than females but larger values for females on the positive latent trait than males. This finding suggested that males and females respond differently to the scale, although the extent of differences may not be problematic. This has not been reported in the literature and can be considered unique. These results could be due to the characteristics of the respondents in the sample or a gender difference in the interpretation of the translated wording of the scale. As such, it is worthy of further examination in future studies. In terms of the impact of gender differences on the validity of the scale, given the small extension of these differences, there may not be a very significant bearing. However, there could be an implication to the utility of the scale for assessing the mental well-being of the general adult population. The study results suggested that the scale could be slightly more sensitive in measuring the mental well-being of males than females. Hence, users of the scale should take note of the possible gender differences in the result of the assessment. Using the standardized conversion score suggested by the original authors could probably help in interpreting the test results. Future validity studies could be conducted to further examine the gender differences in the responses to the scale.

Some limitations have been identified in this study. First, is the skewed gender and age distributions of the sample. The sample consisted of nearly 70% of females with 45% of respondents in the age group 18-34 years. These reflected some gender and age imbalance in sampling. This may pose some limitations to the generalizability of the results obtained. Second, although it is not the aim of the current study to implement a full Classic Test Theory validation study, there is a lack of data using other instruments in assessing a similar construct of mental well-being, such as WHO-5 and PHQ-9, in this study. As such, the convergency validity of the scale cannot be fully examined. In future validation studies, a more gender and age-balanced sample should be used to ensure the generalizability of results. Moreover, it is prudent to include other measures of mental well-being for a more comprehensive examination of the validity of the scale.

In conclusion, the psychometric properties of the Chinese version of the SWEMWBS have been further examined using the Classic Test Theory and the Item Response Theory approaches. Results indicated that as a single-factor scale, on both the scale and item levels, the quality of the instrument is good, with high discriminative power and an adequate response set for assessing a full range of the latent trait, namely mental well-being.

## Data Availability

The raw data supporting the conclusions of this article will be made available by the authors, without undue reservation.
