# Supplementary material for: Further validation of the Chinese short Warwick Edinburgh mental wellbeing scale in the adult population of Macau: an application of classic test theory and item response theory
Source: Front Psychiatry. 2025 Feb 26;16:1528509. doi: 10.3389/fpsyt.2025.1528509 (PMC11897500; doi:10.3389/fpsyt.2025.1528509)
Supplement: Supplementary file 1 [file SupplementaryFile1.docx]

Supplementary Information

Supplementary Figure 1. Test Characteristics Curves

Supplementary Figure 2. Category Characteristics Curves of each item by sex

Supplementary Figure 3. Item Information Functions by sex

Supplementary Figure 4. Test Characteristics Curves by sex

Supplementary Table 1. The range of discriminant parameters and their interpretation

| Discrimination Values (a) | Interpretation |
| --- | --- |
| a > 1.70 | Vert high discrimination; the item functions well |
| 1.35 < a > 1.69 | High discrimination; the item is good no revision is necessary |
| 0.65 < a > 1.34 | Moderate discrimination; the item is fine and needs little revision |
| 0.35 < a > 0.64 | Low discrimination; the item is marginal and needs revision |
| a < 0.34 | Vert low discrimination; the item should be eliminated or revised |

Reference: Baker, F. (2001). The basics of item response theory. ERIC Clearinghouse on Assessment and Evaluation, University of Maryland College Park, MD.
